# Micelles Based on Lysine, Histidine, or Arginine: Designing Structures for Enhanced Drug Delivery

**DOI:** 10.3389/fbioe.2021.744657

**Published:** 2021-09-27

**Authors:** Li Xie, Rong Liu, Xin Chen, Mei He, Yi Zhang, Shuyi Chen

**Affiliations:** School of Medicine and Nursing, Chengdu University, Chengdu, China

**Keywords:** micelles, lysine, histidine, arginine, basic amino acids, drug delivery

## Abstract

Natural amino acids and their derivatives are excellent building blocks of polymers for various biomedical applications owing to the non-toxicity, biocompatibility, and ease of multifunctionalization. In the present review, we summarized the common approaches to designing and constructing functional polymeric micelles based on basic amino acids including lysine, histidine, and arginine and highlighted their applications as drug carriers for cancer therapy. Different polypeptide architectures including linear polypeptides and dendrimers were developed for efficient drug loading and delivery. Besides, polylysine- and polyhistidine-based micelles could enable pH-responsive drug release, and polyarginine can realize enhanced membrane penetration and gas therapy by generating metabolites of nitric oxide (NO). It is worth mentioning that according to the structural or functional characteristics of basic amino acids and their derivatives, key points for designing functional micelles with excellent drug delivery efficiency are importantly elaborated in order to pave the way for exploring micelles based on basic amino acids.

## Introduction

In the past few decades, micelles, as an effective drug delivery system, have considerably attracted worldwide attention for the treatment of tumors. As drug carriers, micelles have many advantages, such as easily synthesizing and modifying chemical structures, nanoparticulate size, enhanced water solubility of drug, prolonged circulation time, increased accumulation in tumor sites, reduced side effects of drugs, as well as improved drug bioavailability and efficiency (Kim et al., 2008; Sun et al., 2009; Aw et al., 2011; Wang et al., 2011). However, up to now, only a few micellar products have been approved by the Food and Drug Administration (FDA) (Cabral and Kataoka, 2014; Deshmukh et al., 2017; Adityan et al., 2020). One possible reason is their potential toxicity (Gupta et al., 2015; Knudsen et al., 2015; Deshmukh et al., 2017). Thus, natural or synthetic biodegradable materials are utilized for constructing micelles to solve the problem (Deng et al., 2012; Grossen et al., 2017; George et al., 2019).

Amino acids are a class of small-molecule compounds that are widespread in nature. More than 300 natural amino acids have been found, but only 20 amino acids take part in the human protein synthesis (Canfield and Bradshaw, 2019; Kelly and Pearce, 2020). Most of the 20 amino acids are good raw materials for fabricating micelles, for example, lysine (Itaka et al., 2003; Cheng et al., 2021; Kanto et al., 2021), arginine (Yao et al., 2016; Jiao et al., 2019), histidine (Guan et al., 2019; Wang Z. et al., 2019), glutamic acid (Krivitsky et al., 2018; Ma et al., 2020; Brunato et al., 2021), aspartic acid (Yeh et al., 2013; Teng et al., 2017), cysteine (Xu W. et al., 2015). Among them, there are three basic amino acids namely, lysine, histidine, and arginine, whose side chains contain amino, imidazolyl, and guanidine groups, respectively. The three basic groups can be protonated in acidic condition to play some special roles in the construction of micelles. For example, amino groups in the lysine side chain can be applied as a chemical attachment site, which facilitates the construction of micelles (Liu et al., 2015). The imidazolyl group of histidine has the characteristics of protonation and deprotonation, making histidine-based micelles pH-responsive (Augustine et al., 2020a). Guanidine group of arginine is positively charged after protonation and contributes to the membrane-penetration ability of micelles (Geihe et al., 2012; Hu et al., 2015). In addition, poly(amino acids) synthesized by the above three basic amino acids have good biocompatibility and biodegradability. Moreover, poly(amino acids) contain many chemically modifiable side groups, providing abundant active groups for constructing functional micelles (Xu H. et al., 2015; Augustine et al., 2020b). Therefore, basic amino acids have a broad application in the field of drug delivery.

This review describes the design and construction of drug-loaded micelles based on the three basic amino acids or their derivatives from the following perspectives: 1) Design of micelles based on the position and role of lysine in the micelle skeleton. 2) Construction of micelles based on the pH-sensitive properties of histidine. 3) Introduction of micelles using arginine’s cell-membrane-penetrating activity, and antitumor ability to convert to NO.

## Lysine-Based Micelles

### Lysine-Based Linkers

Lysine, lysine-based dendrimer, or polylysine may serve as a linker in micelles. Compared with a single lysine molecule as a linker, the lysine-based dendrimer and polylysine possess more abundant active groups.

#### A Single Lysine Molecule as Linker

Lysine contains two amino groups and one carboxylic group. Therefore, when lysine is adopted as a linker during the construction of micelles, it can react with drugs, compounds, or polymeric materials containing active groups.

There are two methods of linking lysine and the above materials: 1) two amino groups of lysine are utilized to connect materials with carboxylic groups. For example, two amino groups of lysine are separately connected with carboxyl group of hydrophobic stearic acid and the terminal carboxyl group of hydrophilic polyglutamic acid to obtain amphiphilic diblock micelle skeleton (Chang et al., 2017); 2) amino group and carboxyl group of lysine are linked to hydrophilic or hydrophobic materials with carboxyl group or amino group. For example, Wang et al. (2012) connected carboxyl group of lysine with hydroxyl group of hydrophilic polyethylene glycol by an ester bond and connected two amino groups of lysine with two vitamin E succinate molecules as hydrophobic anticancer drug to construct a drug-loaded micelle skeleton.

#### Lysine-Based Dendrimer as Linker

**FIGURE 1:**
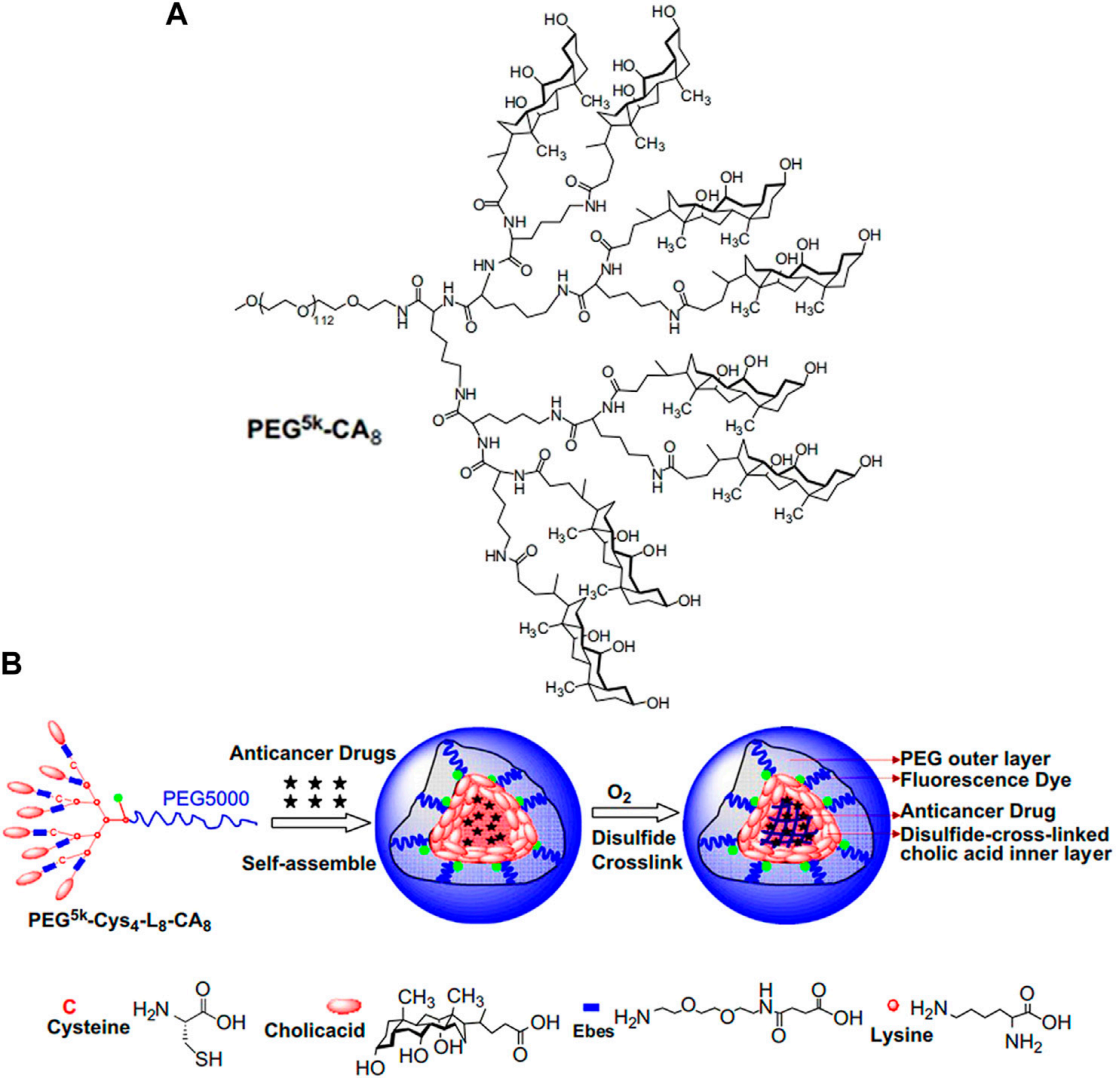
**(A)** The chemical structure of PEG^5k^-CA_8_ G3 lysine dendrimer (Xiao et al., 2009). **(B)** Schematic representation of the disulfide cross-linked micelles formed by the oxidization of thiolated PEG^5k^-Cys_4_-L_8_-CA_8_ after self-assemble (Li et al., 2011). Abbreviation: PEG, polyethylene glycol; CA, cholic acid; Cys, cysteine; L, lysine.

Compared with lysine, lysine-based dendrimer as linker contains more active functional groups, which can provide more conjugation sites for micelles construction (Xiao et al., 2009; Li et al., 2011; Guo et al., 2020). For example, the third-generation lysine dendrimer has one carboxyl group and eight amino groups. In Figure 1A (Xiao et al., 2009), Kit S Lam group built the hydrophilic part of micelles by condensation of carboxyl group of generation 3 (G3) lysine dendrimer and amino groups of monomethyl-terminated polyethylene glycol monoamine with a molecular weight of 5,000 Da (PEG^5k^). Subsequently, eight cholic acid molecules (CA) were connected to the terminal amino groups of G3 lysine dendrimer through an ester bond to construct amphiphilic block polymer (PEG^5k^-CA_8_). Finally, PEG^5k^-CA_8_ was self-assembled into micelles, which significantly increased the paclitaxel loading capacity and fulfilled enhanced *in vivo* drug delivery. Three years later, the same research group made a cross-linked micelle based on PEG^5k^-CA_8_ (Figure 1B; Li et al., 2011). Unlike PEG^5k^-CA_8_, four amino groups of the G3 lysine dendrimer were firstly connected with cysteine (Cys), and then, eight cholic acid molecules were introduced to construct PEG^5k^-Cys_4_-L_8_-CA_8_ micelle. Finally, thiol groups in the cysteine were oxidated to form cross-linked micelles, resulting in the improvement of micellar stability.

#### Poly(L-lysine) as Linker

Poly(L-lysine) has abundant side chain amino groups (Li et al., 2020), which provides many chemical active sites to connect hydrophilic and hydrophobic segments. Therefore, poly(L-lysine) as linker has several advantages over constructing drug or gene delivery vehicle.

**FIGURE 2:**
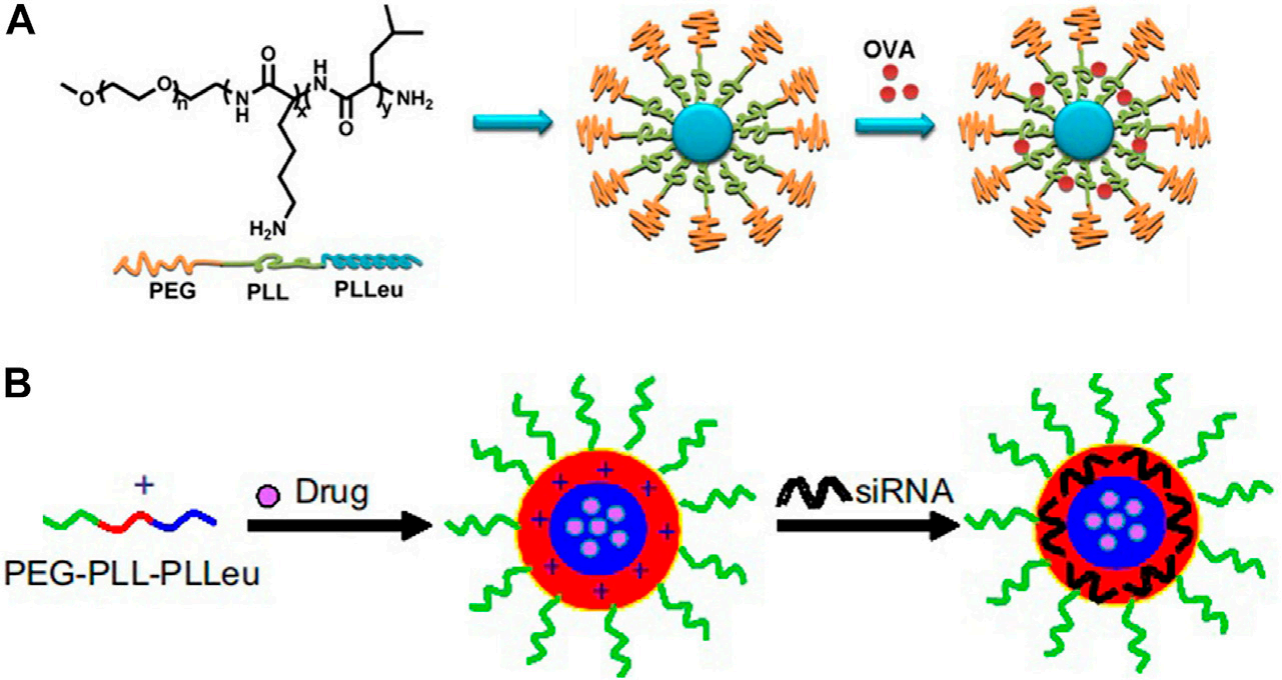
Schematic illustration of PEG-PLL-PLLeu micelles loaded with OVA **(A)** (Luo et al., 2013) or siRNA and drug **(B)** (Zheng et al., 2013). Abbreviation: PEG, poly(ethylene glycol); PLL, poly(L-lysine); PLLeu, poly(L-leucine); OVA, ovalbumin.

Firstly, the side chain amino groups located in poly(L-lysine) interact with drugs, siRNA, or other substances through physical interactions to achieve desired loading of micelle, including electrostatic interactions and hydrophobic interactions. For example, Lintao Cai group designed an amphiphilic triblock copolymer poly(ethylene glycol)-*b*-poly(L-lysine)-*b*-poly(L-leucine) (PEG-PLL-PLLeu) to load ovalbumin (OVA) by electrostatic interactions between the side chain amino groups of polylysine and ovalbumin vaccine (Figure 2A; Luo et al., 2013). Using the same triblock polymer, the research team also used the polyleucine block as the hydrophobic core to incorporate docetaxel by the hydrophobic interactions, while the side chain amino groups of polylysine were used to load siRNA by electrostatic interaction to achieve co-loading of anticancer drug and gene (Figure 2B; Zheng et al., 2013).

**FIGURE 3:**
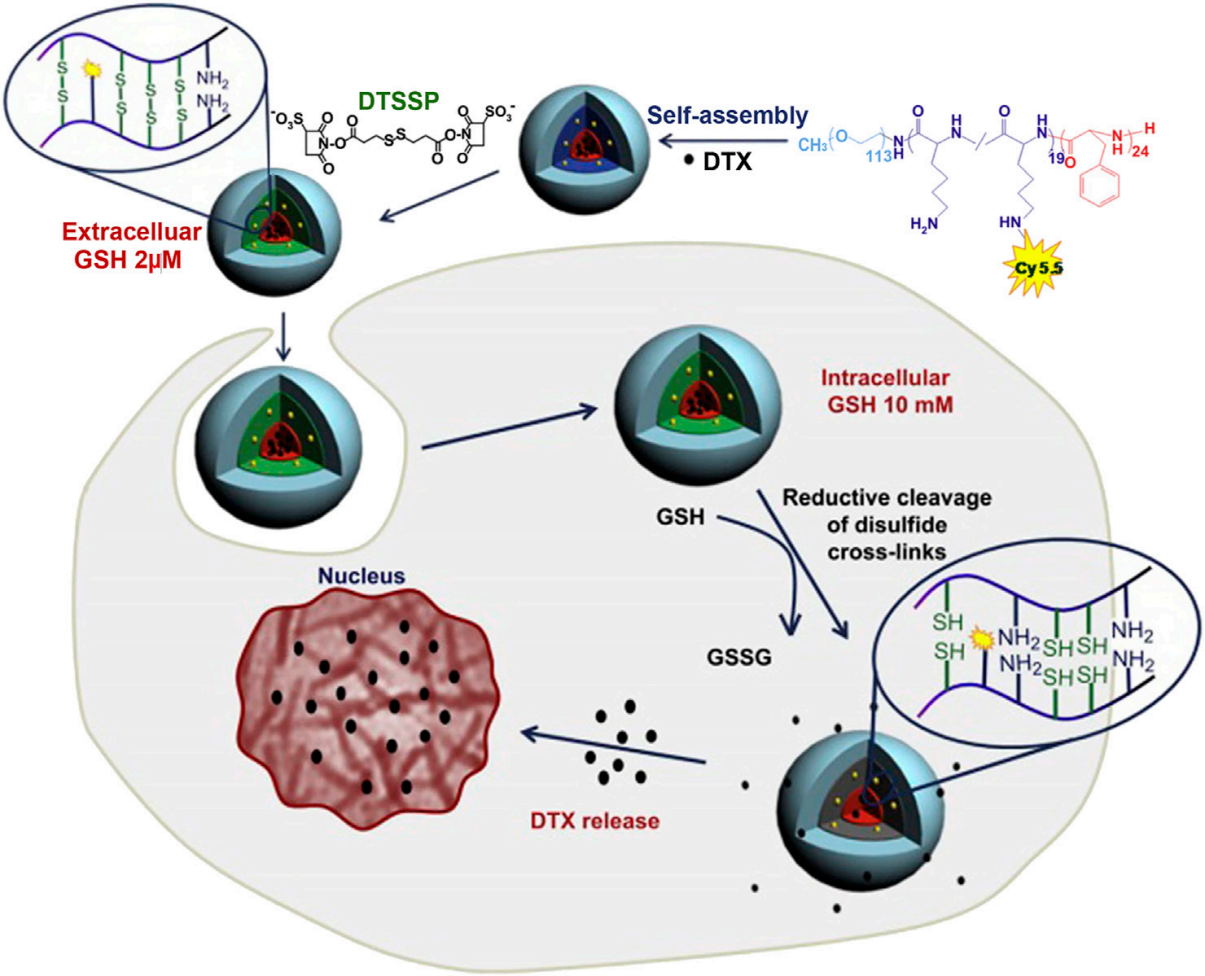
Illustration of shell cross-linking of DTX-loaded micelles with redox-labile disulfide cross-links and triggered release of DTX by intracellular GSH (Koo et al., 2012). Abbreviation: GSH, glutathione; DTSSP, 3,3′-Dithiobis (sulfosuccinimidylpropionate); DTX, docetaxel.

Secondly, covalently cross-linked polylysine linker could be constructed by the addition of disulfide cross-linker to realize the improvement of micellar stability. To take an example, Koo et al. prepared a triblock polymer, poly (ethylene glycol)-b-poly(L-lysine)-b-poly(L-phenylalanine) (PEG-PLys-PPhe) (Koo et al., 2008; Koo et al., 2012), which self-assembled into micelles. Then, the side chain amino groups of polylysine linker were reacted with 3,3′-dithiobis (sulfosuccinimidylpropionate) cross-linkers to realize micellar cross-linking (Figure 3; Koo et al., 2012). For the cross-linked micelles, there was no change in the particle size and spherical morphology. The stability of micelles in solution was investigated by detecting the scattering light intensity. The non-cross-linked and cross-linked micelles were separately incubated in PBS with 50% serum for 30 min, and the scattered light intensity of the former decreased to 49% of the initial intensity, while that of the latter dropped about 5%. Even if the incubation time of the latter was prolonged to 2 h, 85% of the initial intensity was still maintained. In addition, the non-cross-linked and cross-linked micelles were also incubated with a 2.5 g/L SDS solution. At 2 h, the scattering light intensity of the non-cross-linked micelles decreased to 50%, while that of the cross-linked micelles still reached more than 90% of the initial scattering intensity. The above results suggested that micellar stability was improved by cross-linking of polylysine linker. More importantly, disulfide bonds in cross-linked micelles are redox-sensitive, which can be cleaved by glutathione (GSH) (Zhang et al., 2016; Kang et al., 2018). In the blood circulation and cells, there are different concentrations of GSH (Jones et al., 1998; Kang et al., 2018), which directly affected the degree of disulfide bond cleavage in polylysine linker. For example, in the blood circulation, GSH concentration is low (∼2 μM) (Jones et al., 1998), and the degree of disulfide bond breakage is low, resulting in slow drug release in blood. Conversely, the concentration of GSH in most cancer cells is high (∼10 mM) (Kim et al., 2021), the disulfide bond in polylysine linker is broken to a high degree, and the barrier for drug release is caused by the layer of polylysine linker is removed, resulting in faster drug release. Therefore, cross-linked micelles by 3,3′-dithiobis (sulfosuccinimidylpropionate) cross-linkers were beneficial to reduce the loss of encapsulated drugs in the blood circulation and achieve responsive drug release in the target cells.

### Poly(L-lysine) as the Core of Micelles

Poly(L-lysine) is positively charged and water-soluble, containing a lot of amino groups. Theoretically, poly(L-lysine) is difficult to form the core of micelles because of the poor hydrophobicity. However, there are several ways to assist polylysine in constructing the micellar core. First of all, based on electrostatic interaction, polylysine reacts with oppositely charged polyelectrolytes or molecules to significantly reduce its water solubility, so that polylysine can be used to construct the core of micelles (Boudier et al., 2009; Mebarek et al., 2013). In the next place, amino groups of polylysine are chemically modified to enhance the nucleation force of micellar core. In the following sections, the above two ways will be presented separately.

#### Construction of Micellar Core Through Electrostatic Interaction

The positively charged polylysine and negatively charged polymers can facilely form micellar core by electrostatic interaction. Taking polylysine and polymethacrylic acid-*b*-polyethylene oxide as an example, amino group of polylysine and carboxyl group of polymethacrylic acid block were combined by electrostatic interaction, and then, peptide drugs were wrapped to give a stable ternary composite micelle with a particle size of 82 nm (Boudier et al., 2011). The micelle not only kept stable under physiological pH conditions (pH = 7.4) but also facilitated the delivery of peptide drugs. After being endocytosed, the side chain amino group of polylysine was protonated under the acidic condition of cell endosomes (pH = 5), which would disintegrate the micellar core and release peptide drugs.

**FIGURE 4:**
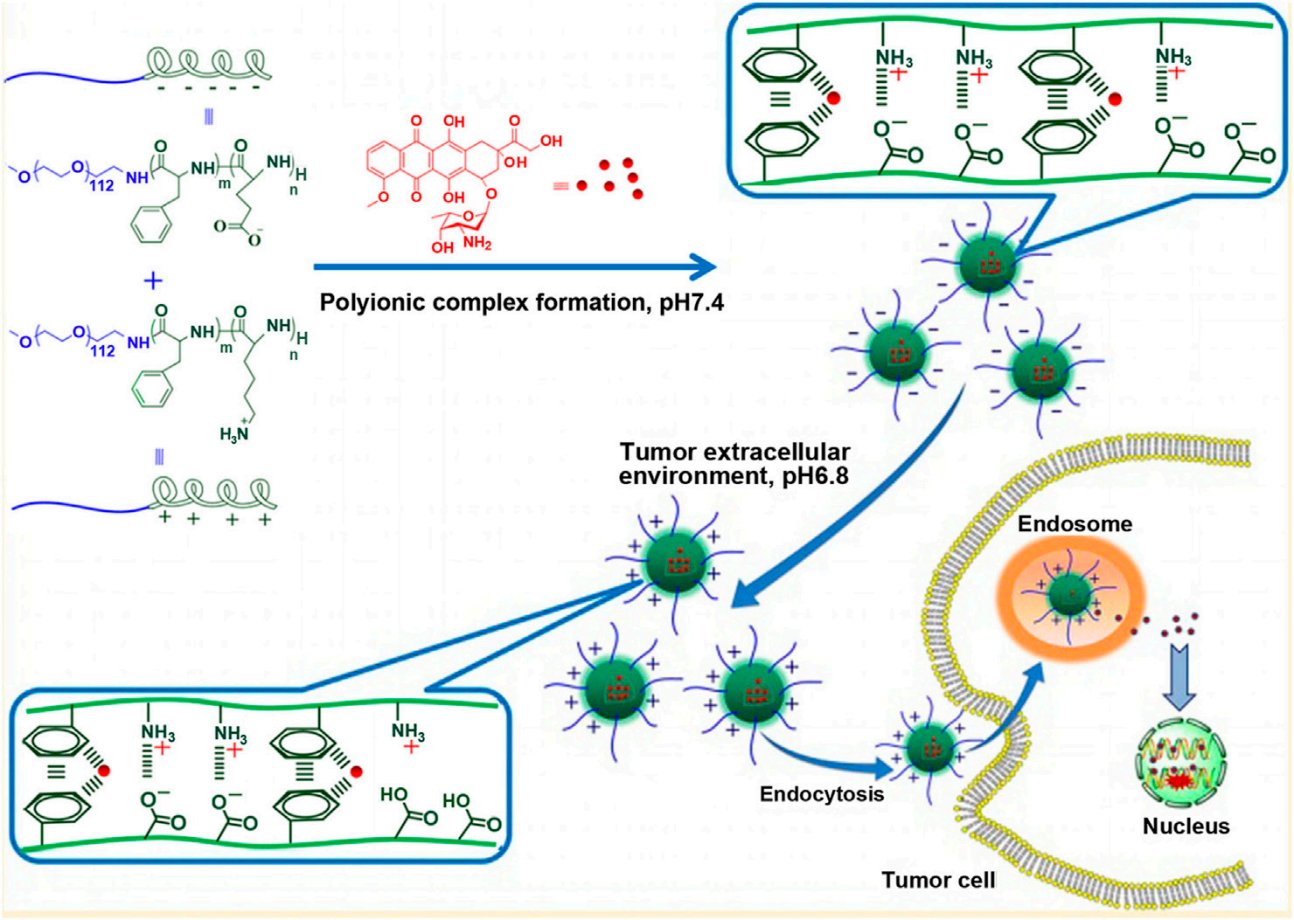
Schematic illustration of charge-conversional behavior and endocytosis performance of polyion complex micelles (Lv et al., 2014).

Alternatively, the terminal group of polylysine is connected with a hydrophilic chain segment, such as polyethylene glycol to form hydrophilic block polymer, which is chosen as positively charged polymer to build the core of the micelle through electrostatic interaction with other hydrophilic block polymers with opposite charge (Xu H. et al., 2015). It has been reported that the influence of the length of positively charged chain of pol(ethylene glycol)-b-poly(L-lysine) and negatively charged chain of poly(ethylene glycol)-b-poly(α, β-aspartic acid) on the self assembly of polyion complex micelles. When the lengths of poly(L-lysine) and poly(α, β-aspartic acid) segments with opposite charge differed greatly, the complex formed by poly (ethylene glycol)-*b*-poly(L-lysine) and poly(ethylene glycol)-*b*-poly(α, β-aspartic acid) was unstable and micelles could not form. For example, there were 18 repeating units of polylysine segment and 78 repeating units of polyaspartic acid segment, or 78 repeating units of polylysine segment and 18 repeating units of polyaspartic acid segment. On the contrary, if there was a small difference in the chain length of poly(L-lysine) and poly(α, β-aspartic acid) segments, micelles with charge would be constructed. While the dissociation degree of carboxyl group and amino group in polymer was easily affected by environmental acidity, resulting pH-dependent charge-reversal micelle was fabricated (Lv et al., 2014). Lv et al. (2014) employed MPEG-NH_2_ as initiator and prepared cationic block copolymer containing lysine and phenylalanine, methoxy poly (ethylene glycol)-b-poly(L-lysine-*co*-L-phenylalanine), by NCA ring-opening polymerization. In the same way, they also prepared anionic block polymer containing glutamic acid and phenylalanine, methoxy poly(ethylene glycol)-*b*-poly(L-glutamic acid-*co*-L-phenylalanine). In two block copolymers, the molar ratio of lysine and L-glutamic acid residues was 1:1.1, that is, lysine repeating unit in cationic block polymer was one less than glutamic acid repeating unit in the anionic block polymer. When methoxy poly(ethylene glycol)-*b*-poly(L-lysine-*co*-L-phenylalanine) and methoxy poly(ethylene glycol)-*b*-poly(L-glutamic acid-*co*-L-phenylalanine) were electrostatically combined to form micelle, due to the presence of free carboxyl residue of glutamic acid, the surface of micelle was negatively charged at physiological pH (pH 7.4). However, in acidic tumor microenvironment, the dissociation degree of carboxyl group decreased, while that of amino group increased. Consequently, the surface charge of micelles reversed from negative to positive, which was not only beneficial for micellar stability under physiological pH but also for their endocytosis after reaching cancer cells, as shown in Figure 4.

Besides adding hydrophilic block polymers with negative charge, some negatively charged photosensitizers, drugs, or genes can also interact with hydrophilic block polymer containing polylysine to form micellar core through electrostatic interactions (Jang et al., 2006; Osada et al., 2010; Dirisala et al., 2014; Lu et al., 2020; Zheng et al., 2020). Positively charged polylysine segments were complexed with negatively charged photosensitizers, genes, or drugs to make them become a part of the micellar core. The encapsulated photosensitizers, genes, or drugs could avoid enzyme degradation or removal by the reticuloendothelial system (Beyermann and Kukula, 2000; Harada-Shiba et al., 2002; Osada et al., 2012), thereby improving the therapeutic effect (Sugisaki et al., 2008; Nishiyama et al., 2009). For example, when poly(ethylene glycol)-*block*-poly(L-lysine) (PEG-PLys) was electrostatically compounded with plasmid DNA (pDNA), pDNA was folded by PEG-PLys to form a hydrophobic rod-like core (Osada et al., 2010). The length of the rod-like core was affected by the length of polylysine segment. The longer the polylysine segment was, the more conducive the DNA was compressed. Moreover, with increasing the length of polylysine, the length distribution of the formed micellar core became narrower, which was favorable for endocytosis (Dirisala et al., 2014).

#### Enhancing the Nucleation Force of Micelles Through Chemically Modifying Amino Groups of Polylysine to Construct Micellar Core

Sulfhydryl groups, carboxyl groups, or hydrophobic molecules are introduced to the side chain amino groups of polylysine segment by chemical method, which improves the nucleation force of micelles containing polylysine segment.

#### Cross-Linking the Micellar Core by Introducing Sulfhydryl Groups

When sulfhydryl groups are introduced into hydrophilic block polymer with polylysine segment, under oxidation condition, sulfhydryl groups are oxidized to form disulfide bond, thereby cross-linking polylysine segment and forming core cross-linked micelle (Miyata et al., 2004; Vachutinsky et al., 2011; Oe et al., 2014; Takeda et al., 2017). Core cross-linked micelles show enhanced resistance to the shear stress of blood circulation. Takeda et al. (2017) integrated 1-imino-4-mercaptobutyl (IM) groups as cross-linking agent into the side chain amino groups of polylysine segment in poly (ethylene glycol)-*b*-poly(L-lysine) (PEG-PLys). Introduction rate of IM accounted for 49% of lysine residues of polylysine segment. Under oxidation condition, PEG-PLys (IM) and pDNA were compounded to form core cross-linked polyplex micelle at the N/P ratio of 2:0, which was defined as a residual molar ratio of amino groups in PEG-PLys to phosphate groups in pDNA. In this process, it was accompanied by disulfide bond cross-linking and a combination of positive and negative charges. When core cross-linked micelles were exposed to venular blood flow (30 dyne/cm^2^ shear stress) for 30 min, they could remain stable, and the size of the rod-shaped core was not affected. In contrast, non-cross-linked micelles showed a size increase, from 194 to 242 nm, and pDNA degradation up to 50% under the same condition. Even if shear stress reached 100 dyne/cm^2^, the particle size of core cross-linked micelles was still maintained unchanged. Maintenance of the particle size was not only beneficial for micelles to enter cells and protect pDNA from degradation by DNasel but also significantly prolonged residence time of pDNA in mice and improved gene transfection efficiency *in vivo*.

#### Charge Reversal of Polylysine by Introducing Carboxyl Groups

**FIGURE 5:**
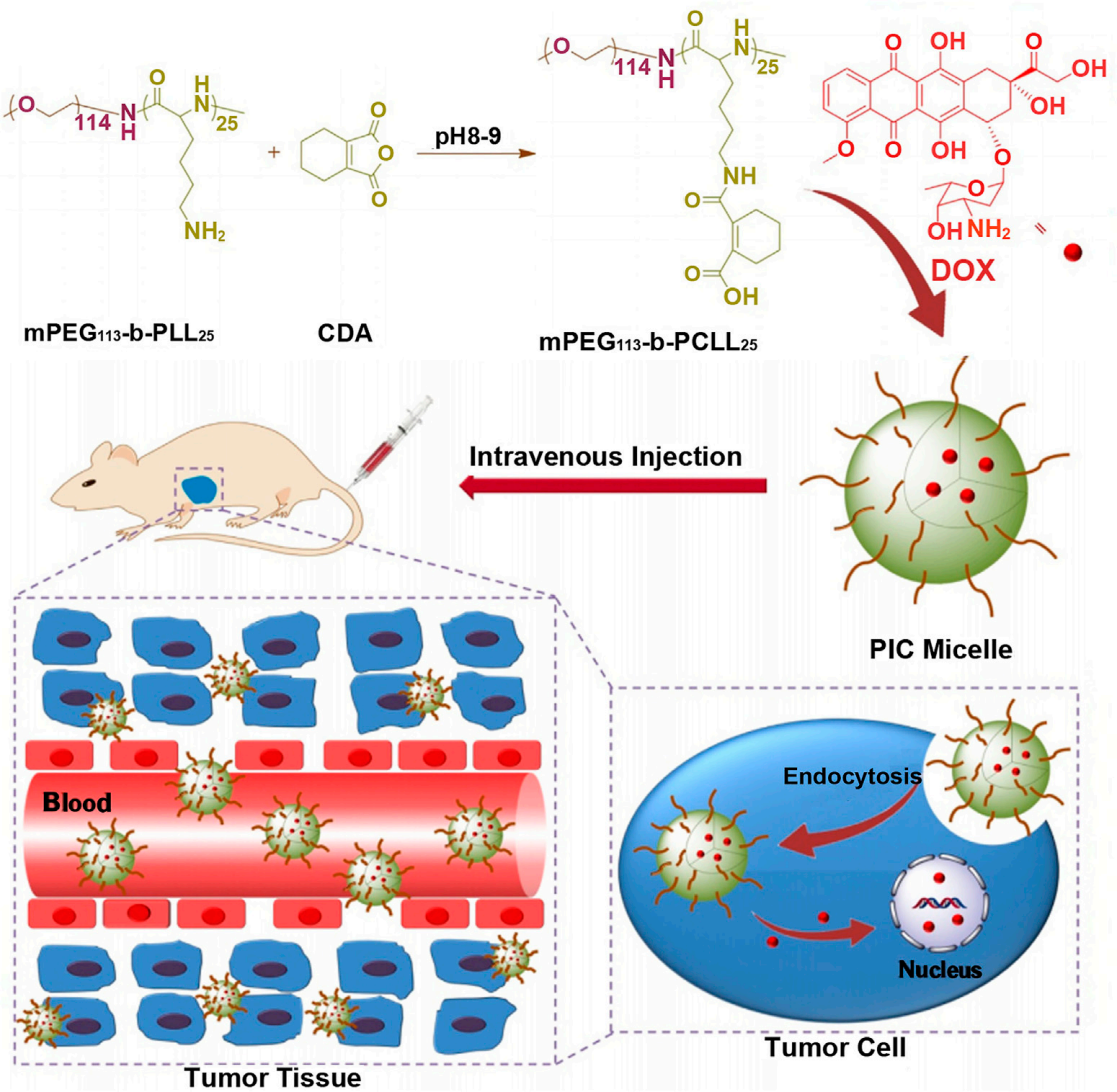
Schematic illustration of the preparation of pH-responsive PIC micelle, and its circulation *in vivo*, accumulation in tumor tissue, and finally, pH-triggered intracellular DOX release after intravenous injection (Wang J. et al., 2015). Abbreviation: PEG, poly(ethylene glycol); PLL, poly(L-lysine); CDA, cis-cyclohexene-1,2-dicarboxylic anhydride; PCLL, poly(N(ε)-((1-carboxy-cis-cyclohexene)-2-carbonyl)-L-lysine; DOX, doxorubicin; PIC micelle, polyion complex micelle.

If carboxyl groups are introduced into polylysine, its electrical property will vary from positive to negative charge. Thus, polylysine with negative charge can be combined with drugs with positive charge, such as doxorubicin with one amino group, to realize the load of positively charged drugs. For example, the ring-opening polymerization of cis-cyclohexene-1,2-dicarboxylic anhydride (CDA) was initiated from the side chain amino groups of methoxy poly(ethylene glycol)-*block*-poly(L-lysine) (mPEG-*b*-PLL) to get anionic methoxy poly(ethylene glycol)-*block*-poly(N(ε)-((1-carboxyl-cis-cyclohexene)-2-carbonyl)-L-lysine (mPEG-*b*-PCLL) with acid-degradable side amide bond, which was shown in Figure 5 (Wang J. et al., 2015). The carboxyl-functionalized polylysine segment of mPEG-b-PCLL interacted with doxorubicin to form micellar core. In acidic extracellular microenvironment of tumor cells (pH = 6.8), the volume of micelles expanded, resulting in rapid release of drug. When micelles entered more acidic endo/lysosomes (pH = 5.5), the cumulative release rate of drug was over 75% at 72 h. This might be related to the cleavage of the side amide bond of mPEGpolypeptide copolymer, leading to the disassembly of the micellar core (Chen et al., 2015).

#### Hydrophobic Modification of Polylysine by Introducing Hydrophobic Molecules

After introducing hydrophobic molecules into the side chain amino groups of polylysine, the solubility of polylysine was changed from hydrophilic to hydrophobic. The modified hydrophobic polylysine could directly construct the core of micelles and wrap hydrophobic drugs (Wen et al., 2011; Ding et al., 2013; Lin et al., 2014). For instance, ε-carbobenzyloxy-L-lysine N-carboxyanhydride (Lys(Z)-NCA) was first synthesized by using N-ε-carbobenzyloxy-L-lysine as starting material. Then, it was reacted with propargylamine through ring-opening polymerization to produce hydrophobic α-alkyne-poly-(N-ε-carbobenzyloxy-L-lysine) (α-alkyne-PZLL). Next, α-alkyne-PZLL was added to further link with dendritic polyamidoamine (PAMAM) block copolymer (N_3_-D3) to synthesize amphiphilic PZLL-block-dendritic PAMAM copolymers (PZLL-D3) by copper-catalyzed azidealkyne cyclization (Lin et al., 2014). PZLL-D3 micelles enabled co-loading of doxorubicin and gene, where the PZLL core loaded doxorubicin through hydrophobic interactions, and the PAMAM shell carried gene through electrostatic interactions.

**FIGURE 6:**
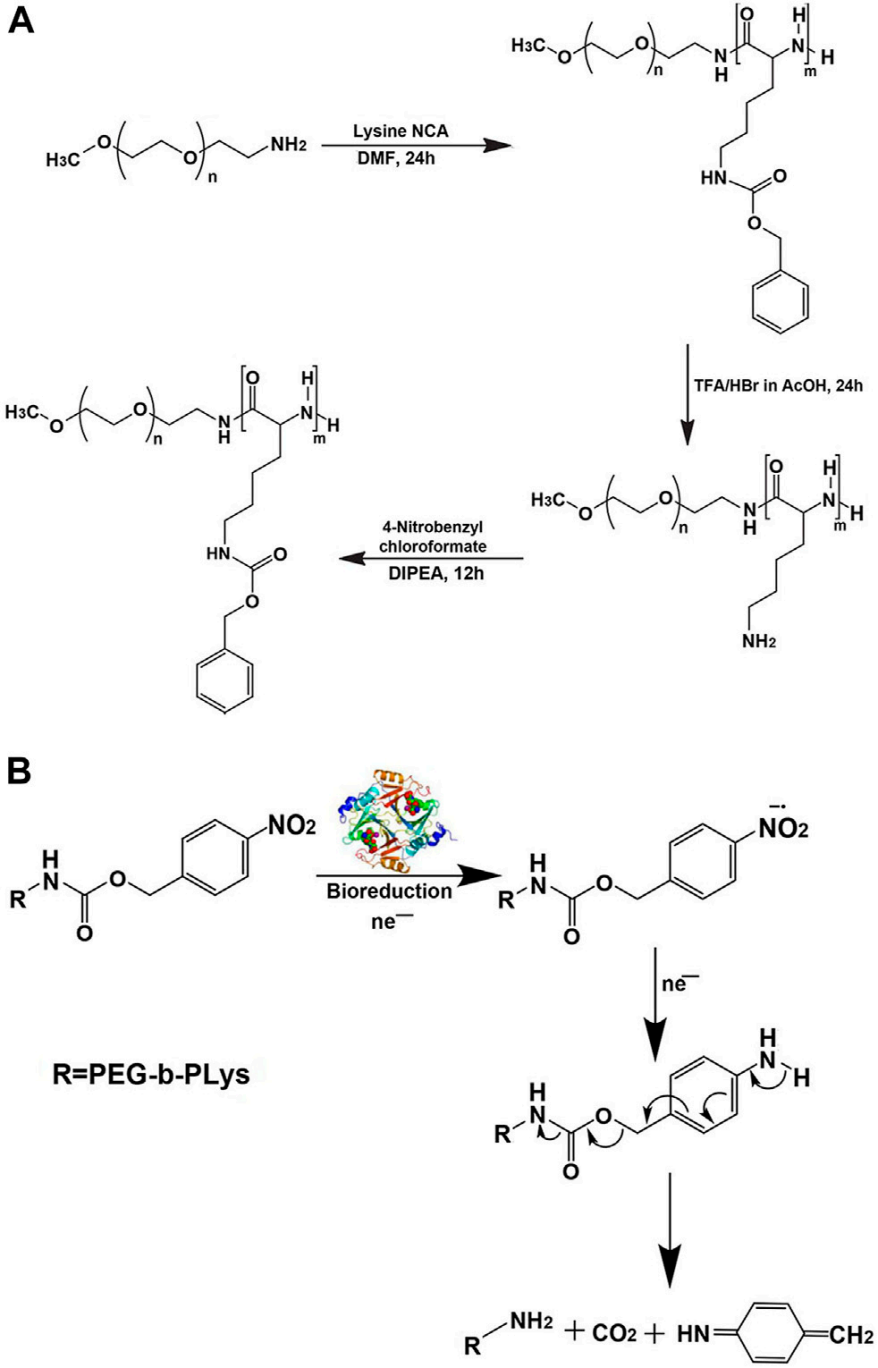
Synthetic route for preparation **(A)** and mechanism of hypoxic reduction under the nitroreductase of the hypoxia-sensitive copolymer **(B)** (Thambi et al., 2016). Abbreviation: PEG, poly(ethylene glycol); PLys, poly(L-lysine); DIPEA, N,N-diisopropylethylamine.

If nitro group was also introduced into the para-position of benzene ring of poly-(N-ε-carbobenzyloxy-L-lysine) segment of micelle, it would make micelle sensitive to hypoxia, which was more conducive to the rapid drug release of micelle in the hypoxic tumor tissue (Thambi et al., 2016). The case in point was the two-layered biodegradable micelle prepared by self-assembly of poly(ethylene glycol)-*b*-poly(ε-(4-nitro)benzyloxycarbonyl-L-lysine) (PEG-*b*-PLys-*g*-NBCF) diblock copolymers (Figure 6A). Under the hypoxic environment, the nitro groups on NBCF were easily reduced to produce amine groups by a series of six electron-transfer reactions in the presence of cellular nucleophiles or nitroreductases. As a consequence, the formed amine groups continued to induce the degradation of the N-(4-aminobenzyloxycarbonyl)-based derivative into small molecular compounds. Owing to the loss of hydrophobic NBCF groups in the polylysine segment of micelle, the micellar core disintegrated and drug was rapidly released (Figure 6B).

### Polylysine as Micellar Shell

When polylysine is connected to hydrophobic block, polylysine will act as the shell of micelle so that the surface of micelle is positively charged. However, it is generally known that cationic nanocarriers interact strongly with serum components, causing severe aggregation and rapid clearance by the reticuloendothelial system (RES) (Oupicky et al., 2002; Zhang et al., 2020), which results in their short half-life in the blood circulation.

**FIGURE 7:**
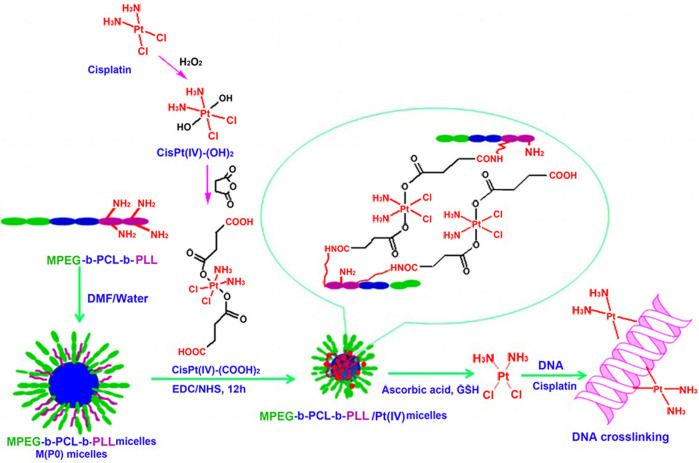
Preparation of MPEG-b-PCL-b-PLL/cisPt (IV) micelles and the proposed pathway of action (Song et al., 2013). Abbreviation: PLys, poly(L-lysine); PLLA, poly(L-lactide); PIC, polyion complex.

Researchers have made some efforts to address the above issues. A good strategy is to shield positive charge of micelles by occupying amino groups of polylysine. Song et al. (2013) cross-linked polylysine chains in the micellar shell by introducing cisplatin (IV) prodrug as a bi-functional cross-linker to reduce the number of free amino groups. As a result, the amount of positive charge on the surface of micelles was reduced. As shown in Figure 7, monomethoxyl poly(ethylene glycol)-b-poly(ε-caprolactone)-*b*-poly(L-lysine) (MPEG-*b*-PCL-*b*-PLL) block copolymer self-assembled into micelle, whose shell was composed of MPEG and PLL segments. Thereafter, PLL segment was cross-linked by cisplatin (IV) prodrug, and amino groups of PLL segment were occupied, which reduced the surface positive charge of micelles. Moreover, the larger the proportion of cross-linked amino groups was, the lower the positive charge of micelles was. The most important thing to note from Figure 7 was the triggered release of cisplatin (IV) prodrug from the cross-linked micellar shell under acidic condition or in the presence of mild reducing agents. At pH 5.0, the release rate of platinum was significantly higher than that at pH 7.4. Under the condition of 5 mM sodium ascorbate, cisplatin (II) was directly released. The released cisplatin (II) could be chelated by DNA nucleobases of tumor cells, which led to the cross-linking between adjacent nucleobases, along with preventing DNA replication, transcription, and cell division (Boulikas et al., 2007; Khoury et al., 2020).

**FIGURE 8:**
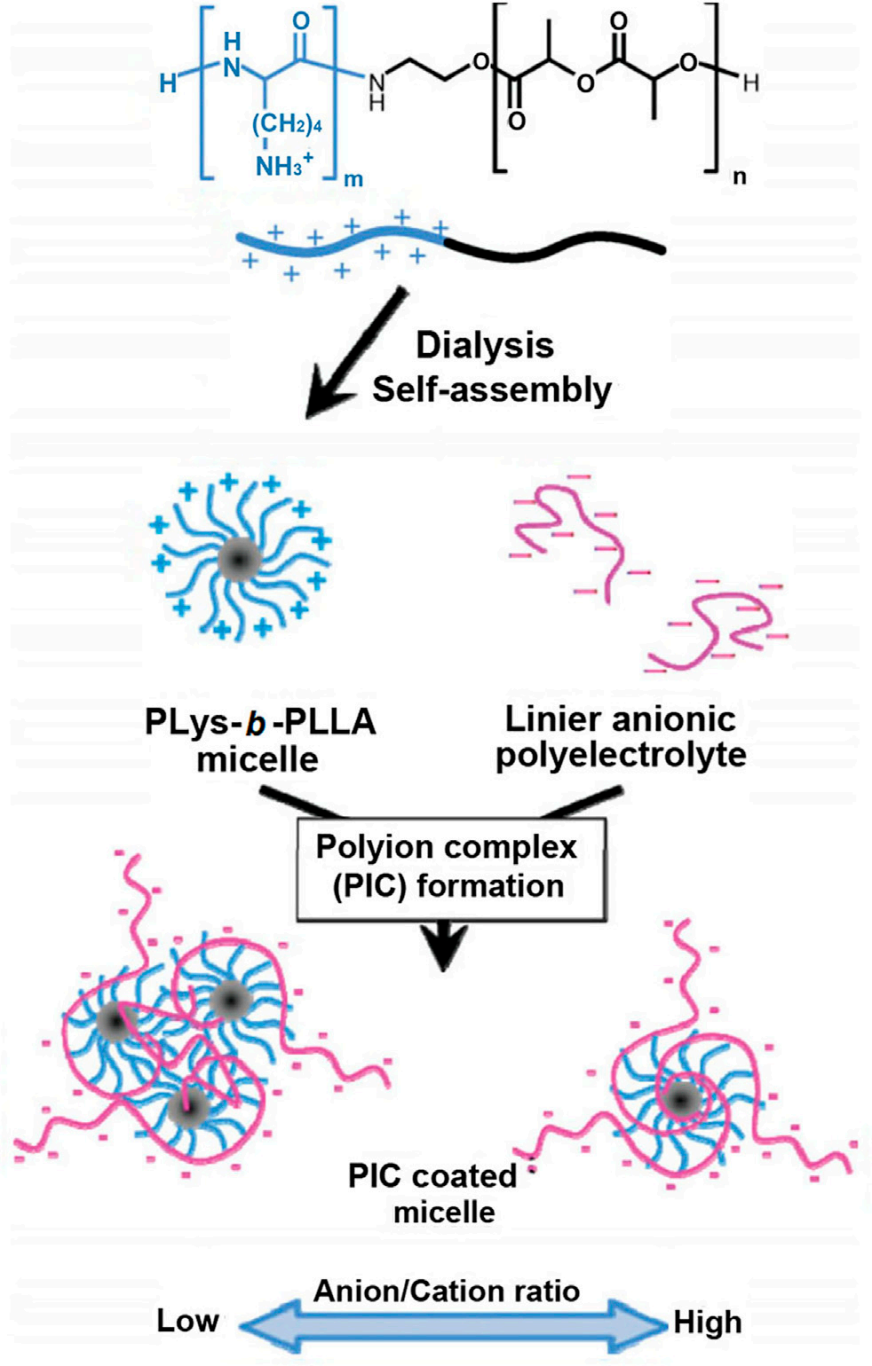
Schematic illustration for the preparation of HA-coated micelles (Ohya et al., 2010). Abbreviation: HA, hyaluronic acid; PLys, poly(L-lysine); PLLA, poly(L-lactide).

Another example was shown in Figure 8 (Ohya et al., 2010). Anionic hyaluronic acid (HA) was coated on the outer layer of poly(L-lysine)-*b*-poly(L-lactide) (PLys-*b*-PLLA) block copolymer micelles by electrostatic interactions. Because amino groups of polylysine were covered by HA, the HA-coated polylysine micelles possessed extremely high resistance to dilution and colloidal stability, whose apparent CMC was 2.1 × 10^−11^ mg/ml and hydration particle size remained stable in PBS solution containing 10% FBS for 24 h. In the follow-up study (Ohya et al., 2011), the polyanion micelle was used for drug delivery. HA not only improved the stability of micelles and prolonged circulation time in the blood but also controlled drug release rate and reduced the cytotoxicity of HA-coated polylysine micelles.

**FIGURE 9:**
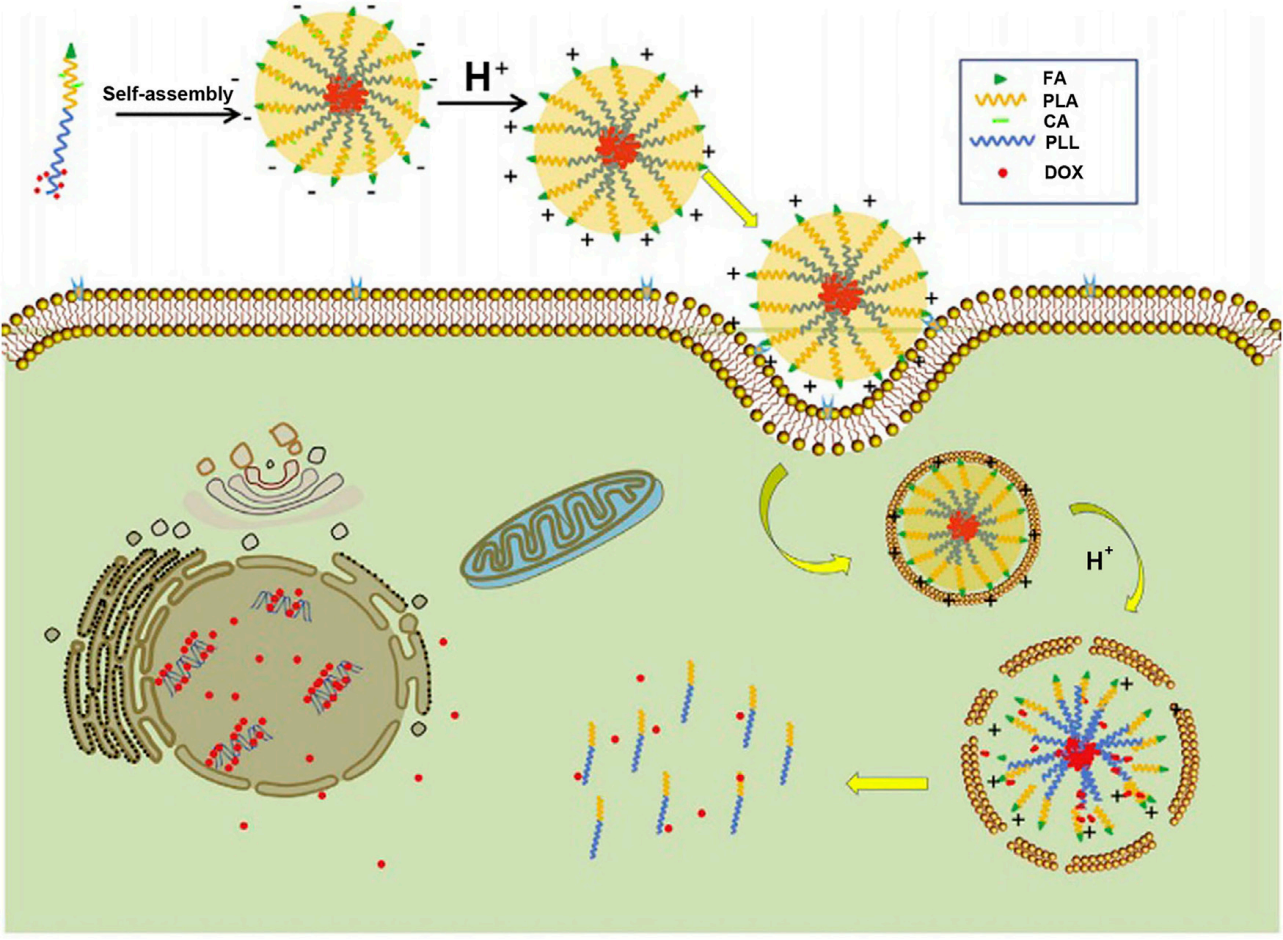
Illustration of the folate-conjugated pH-responsive polymeric micelles based on FA–PLL(CA)–PLA for receptor-mediated endocytosis and pH-triggered release (Guan et al., 2017). Abbreviation: FA, folate; PLL, poly(L-lysine); CA, citric acid; PLA, poly(lactc acid); DOX, doxorubicin.

“Constructing charge-reversal polylysine segment” is another strategy to solve the problem of cationic micelles-based polylysine. The micelles based on charge-reversal polylysine are negatively charged in the blood circulation and will complete positive-surface conversion after reaching tumor tissue or cells (Du et al., 2010; Zhang et al., 2020), which is beneficial to improve the phagocytosis of tumor cells (Du et al., 2010; Pittella et al., 2011). Guan et al. (2017) synthesized folate-poly(L-lysine)-poly(lactic acid) (FA-PLL-PLA), and then, citric acid (CA) was introduced into the side chain amino groups of polylysine by amide bond to complete the construction of a target copolymer, FA-PLL(CA)-PLA. FA-PLL(CA)-PLA could self-assemble to form micelles, whose surface charge was −19.1 mV at pH 7.4. Under the acid environment (pH = 6.5), the micelles were positive in charge (+15.5 mV). Furthermore, the positive charge rapidly increased as the pH value decreased (Figure 9). The negative-to-positive charge reversal of micelles is due to break of the amide bond between CA and the side chain amino groups of polylysine segment under the acidic condition. The design of charge-reversal polylysine segment could mask the positive charge of micelles in the blood circulation and expose the positive charge in the tumor tissue or cells, which is beneficial to keep the micellar stability under physiological pH and increase the binding opportunities of the micelles with negatively charged tumor cell membrane in the acidic tumor microenvironment.

## Histidine-Based Micelles

Extracellular pH of most solid tumors is about 5.8–7.2 (Lee et al., 2008), which is mainly due to lactic acid derived from anaerobic glycolysis and carbonic acid formed from carbon dioxide and water by carbonic anhydrase over-expressed in tumor (Fukumura and Jain, 2007; Lee et al., 2008). Materials protonated in this pH range are suitable for building pH-sensitive drug-delivery systems to target tumor. Histidine is an excellent candidate, whose pK_a_ value is about 6.0. Moreover, histidine is the only amino acid containing imidazole ring among 20 natural amino acids, which has an electron lone pair on the unsaturated nitrogen, resulting in the amphoteric formation of histidine by protonationdeprotonation (Wu et al., 2013). In the weak acid environment, the imidazole ring undergoes protonation, which making transform of histidine from hydrophobic to hydrophilic, resulting in the increasing solubility of histidine (Liu et al., 2012b). Thus, it is a good method for constructing pH-sensitive micelles to introduce single histidine or polyhistidine into micelles.

### Construction of pH-Sensitive Micelles Using Single Histidine

The simplest way to develop micelles containing histidine is to attach hydrophobic histidine to hydrophilic macromolecules, such as hyaluronic acid (Wu et al., 2016), dextran (Yin et al., 2020), and auricularia auricular polysaccharide (AAP) (Wang et al., 2017). In the above micelles, histidine is usually used as a micellar core. Taking histidine-modified AAP (His-AAP) as an example, histidine was linked to AAP by an ester bond. In a neutral medium, His-AAP self-assembled into micelles. Histidine and AAP acted as hydrophobic core and hydrophilic shell, respectively. Next, paclitaxel was loaded into His-AAP micelles. Compared to that at pH 7.4, the paclitaxel accumulated release rate was increased by about 18% for 12 h at pH 5.0 (Wang et al., 2017). Research by Chang et al. (2010) also confirmed that micelles based on histidine had the characteristic of pH-sensitive drug release. N-Boc-histidine was made use of capping PLGA-PEG-PLGA triblock copolymer to prepare histidine-PLGA-PEG-PLGA-histidine. Then, capping triblock polymer self-assembled into micelles. The drug cumulative release rate of DOX-loaded micelles was approximately 20% higher in pH 6.2 medium than that in pH 7.4 medium at 12 h. The elevated accumulative release rate was mainly associated with instability of micellar core, which was caused by ionization of imidazole ring in histidine-PLGA-PEG-PLGA-histidine triblock polymer. In addition, the cumulative release rate of drug-loaded micelles based on PLGA-PEG-PLGA without histidine modification did not differ significantly at pH 6.2 or 7.4, confirming that histidine played a key role in pH-sensitive capacity of micelles.

### Construction of pH-Sensitive Micelles With Polyhistidine

Different from histidine, polyhistidine can construct both pH-sensitive middle layer and core of micelles. Furthermore, how to regulate pH-responsiveness of micelles is an important factor needed to be focused on. Although polyhistidine was used as either an intermediate layer or core of micelles, factors affecting pH-responsiveness differed, which would be introduced separately below.

#### Factors Affecting pH Response of Micelle When Polyhistidine is Adopted as the Intermediate Layer of Micelles

**FIGURE 10:**
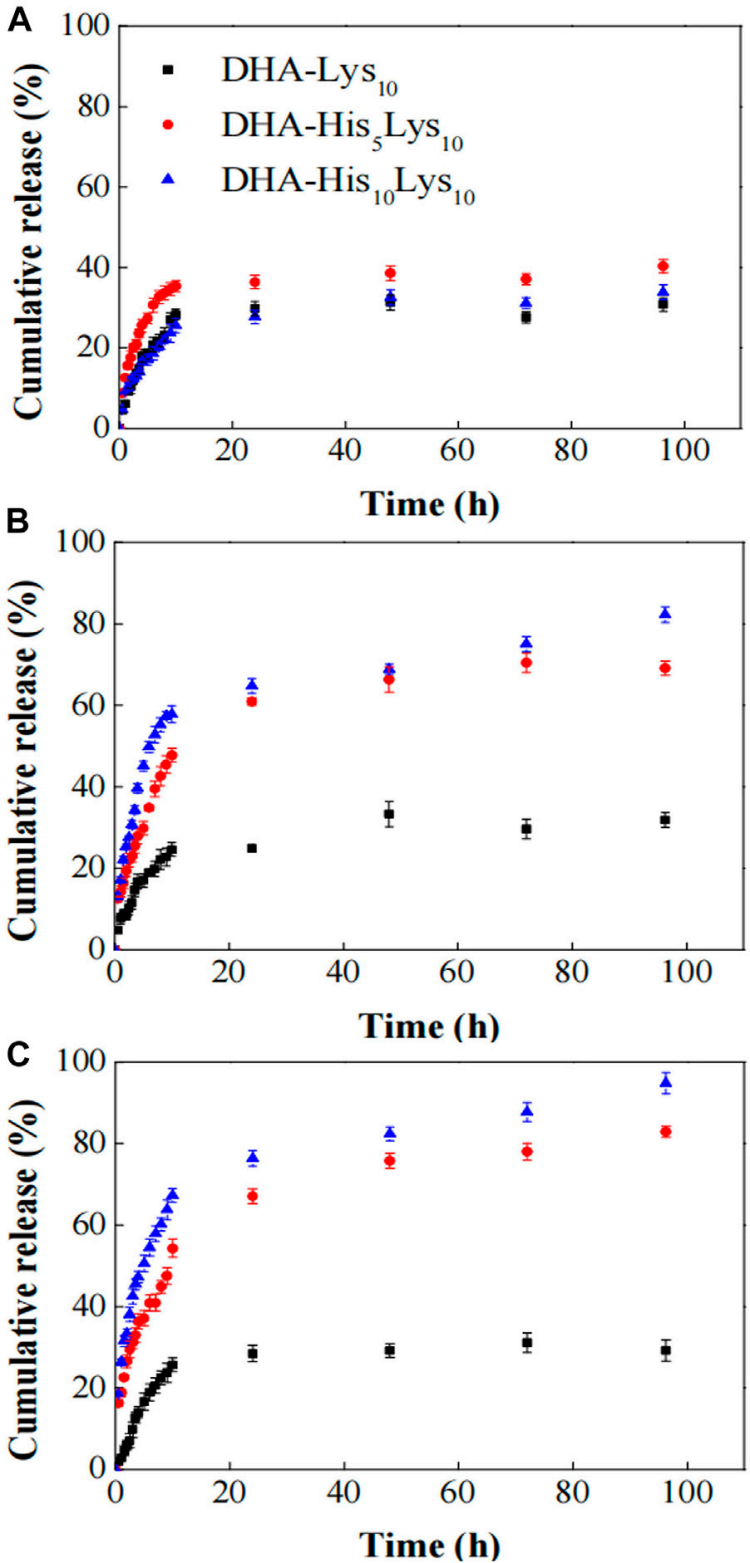
*In vitro* DOX release from DHA-His_x_Lys_10_ (x = 0, 5, 10) micelles at pH 7.4 **(A)**, 6.0 **(B)**, and 5.0 **(C)** PBS solutions (Wang Y. et al., 2015). Abbreviation: DOX, doxorubicin; DHA, docosahexaenoic acid; His, histidine; Lys, lysine.

pH-sensitive capability for polyhistidine as a middle layer of micelles is related to the length of polyhistidine block, namely the number of repeat units of histidine. When the number of repeat units of histidine is relatively small such as 5 and 10, polyhistidine micelles have good capability in pH-sensitive drug release. Therefore, the number of repeat units of histidine is a key factor, which should be considered in the process of designing micelles. In a report, block copolymers Histidine_x_Lysine_10_ (His_x_Lys_10_, x = 0, 5, and 10) conjugated with docosahexaenoic acid (DHA) was designed and prepared to self-assemble into micelles (Wang Y. et al., 2015), in which DHA acted as hydrophobic inner core for DOX loading, polyhistidine served as an intermediate pH-sensitive layer, and polylysine block played as hydrophilic shell. *In vitro* drug release experiments were performed in three PBS solutions of pH 7.4, 6.0, and 5.0. As shown in Figure 10, at pH 7.4, all three micelles, DHA-Lys_10_, DHA-His_5_Lys_10_, and DHA-His_10_Lys_10_, released slowly, and the accumulative DOX release ratio was less than 40% in 100 h. At pH 6.0, DOX release accelerated from both DHA-His_5_Lys_10_ and DHA-His_10_Lys_10_ micelles, which was attributed to protonation of imidazole groups of some histidine residues in micelles, whereas the rate of DOX release from DHA-His_5_Lys_10_ and DHA-His_10_Lys_10_ micelles was further speeded up at pH 5.0, especially in the first 10 h. Those results were most likely because the lower pH made more imidazole rings protonate, resulting in the expansion of micellar structure. Furthermore, the longer the histidine block is, the faster the DOX release rate is. In both pH 6.0 and 5.0 media, DOX release from DHA-His_10_Lys_10_ micelles was faster than that of DHA-His_5_Lys_10_ micelles, and the former micelles had a more complete DOX release within 100 h.

However, with increasing the number of repeat units of histidine in micelles, hydrogen-bonding interactions between histidines are probably enhanced, thereby changing the behavior of pH-sensitive drug release. For example, Liu et al. (2012a) used methyl poly(ethylene glycol)-poly(L-histidine)-poly(L-lactide) (mPEG-PH-PLLA) triple block copolymers to investigate the effect of the length of polyhistidine segment on DOX release. At pH 5.0, the cumulative DOX release of mPEG_45_-PH_15_-PLLA_82_ micelles is close to 80% at 24 h, while for mPEG_45_-PH_30_-PLA_82_, the release rate of DOX was 55% at 24 h, and it could not reach 80% until 76 h. It was possible that stronger hydrogen bond interactions existed in polyhistidine of mPEG_45_-PH_30_-PLA_82_, leading to retarded DOX release, compared with mPEG_45_-PH_15_-PLLA_82_. Therefore, when selected as an intermediate layer of micelles, polyhistidine with appropriate chain length can effectively respond to tumor acidic pH microenvironment.

#### Factors Affecting pH Response of Micelles When Polyhistidine Acts as Micellar Core

**FIGURE 11:**
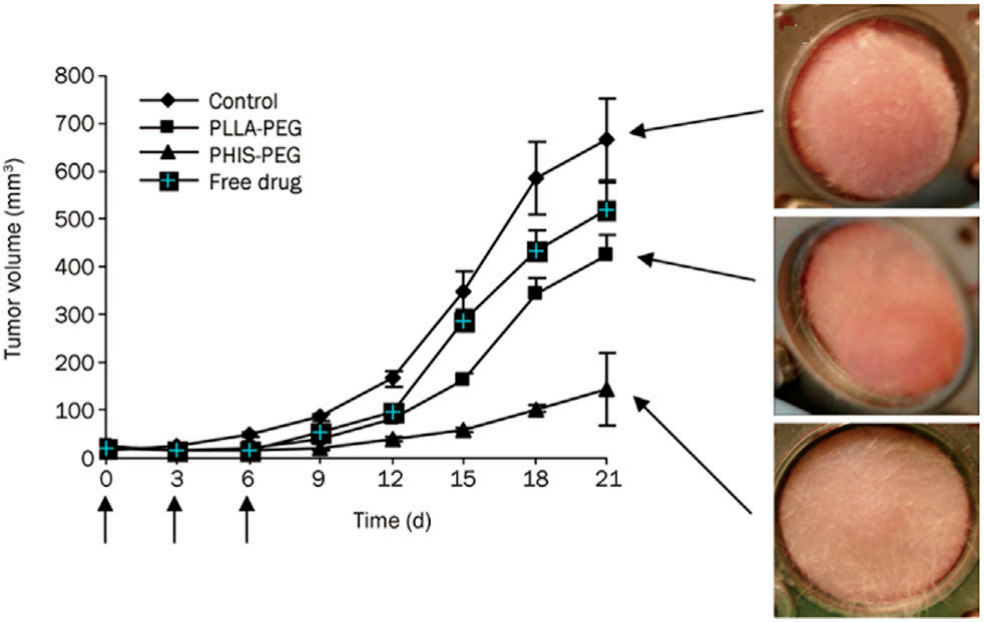
Effects of DOX-loaded pH-sensitive PH-b-PEG micelles and pH-insensitive PLLA-b-PEG micelles on the growth of MDA 231 breast carcinoma in the window chamber model (Jin et al., 2014). Each formulation was administered three times at 3-day intervals (arrows) at a dose of 10 mg/kg for tumor growth inhibition study. Abbreviation: PEG, poly (ethylene glycol); PH, poly(L-histidine); PLLA, poly(L-lactic acid).

There are two methods to regulate pH-responsiveness of micelles when polyhistidine acts as a micellar core. The first method is to connect hydrophilic polymer block to polyhistidine. pK_b_ value of the whole block copolymer is increased due to the introduction of hydrophilic block segments (Mccurdie and Belfiore, 1999). For example, polyhistidine with a molecular weight of 5,000 had a pK_b_ value of about 6.5 (Lee E. S. et al., 2003). After introducing PEG with a molecular weight of 2,000, a PH (*M*_*n*_ 5,000)/PEG (*M*_*n*_ 2,000) block copolymer is formed with a pK_b_ value of about 7.0. As pH is below 7.0, the hydrophilicity of poly(L-histidine) chain segment increased for ionization of histidine imidazole groups, leading to instability of micellar core (Jin et al., 2014). This pH-responsive property is good for drug release outside tumor cells. Jin et al. (2014) adopted pH-responsive PH-*b*-PEG micelles and non-pH-responsive PLLA-*b*-PEG ones to investigate DOX release capability for pH-sensitive micelles based on polyhistidine. In nude mice xenografted by MDA-MB-231 breast cancer, DOX release from micelles was observed by intravital fluorescence microscopy. For PH-*b*-PEG micelles, DOX diffused from tumor blood vessels at a faster rate than that of PLLA-*b*-PEG ones. It was suggested that pH-sensitive PH-*b*-PEG micelles had a rapid dissociation in tumor acidic pH microenvironment, which might reduce the barrier in tumor blood vessels. Moreover, rapid release of DOX made local drug concentration high within the tumor, thus improving bioavailability of drug. After DOX-loaded PH-*b*-PEG and PLLA-*b*-PEG micelles were separately administered *via* tail vein to nude mice three times at 3-day intervals at a dose of 10 mg/kg, the fluorescence intensity of cells was detected *via* flow cytometry, which was extracted from tumor tissues. The result showed that the intensity of cellular uptake of DOX supplied from PH-*b*-PEG micelles was approximately 3.3 times higher than that in PLLA-*b*-PEG micelles. PH-*b*-PEG micelles rapidly released in tumor extracellular pH, which not only promoted endocytosis for drug penetration into tumor cells but also led to more effective inhibition of tumor growth. The tumor volume of PLLA-*b*-PEG micelles treated group was 4.3 times higher than that of PH-*b*-PEG micelles treated group after 21 d of administration, and the latter group significantly suppressed the growth of tumor volume (*p* < 0.01) (Figure 11). Above results fully demonstrated that compared with pH-insensitive micelles, it was able for pH-sensitive PH-*b*-PEG micelles to rapidly disintegrate and trigger drug release in the acidic tumor interstitium.

**FIGURE 12:**
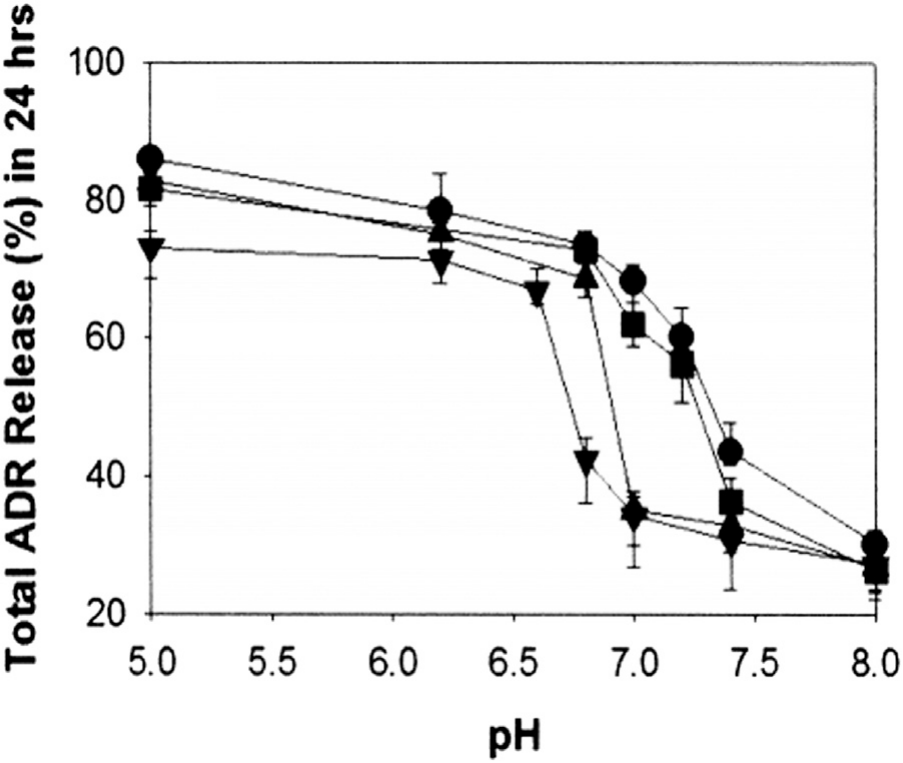
pH-dependent cumulative adriamycin (ADR) release from the mixed micelles composed of polyHis/PEG and PLLA/PEG [PLLA/PEG content in the mixed micelles: 0 wt.% (●), 10 wt.% (■), 25 wt.% (▲), and 40 wt.% (▼)] (Lee E. et al., 2003).

The second approach is that hydrophobic material is introduced into polyhistidine block by blending or copolymerization. It is known that a hydrophobic environment will reduce the local dielectric constant, thereby weakening the ionization tendency of ionizable groups (Benns et al., 2000; Putnam et al., 2001). Thus, hydrophobic material has an inhibitory effect on the ionization of imidazole group in polyhistidine block, which affects the pH value of triggering release of drugs from micelles and modulates the pH-responsiveness of micelles. Moreover, with increasing the content of hydrophobic material, pK_b_ of imidazole groups in polyhistidine block shifts to a lower pH. For example, mixed micelles were prepared by PH (*M*_*n*_ 5,000)/PEG (*M*_*n*_ 2,000) (PH-*b*-PEG) and PLLA (*M*_*n*_ 3,000)/PEG (*M*_*n*_ 2,000) (PLLA-*b*-PEG) block copolymers (Lee E. et al., 2003). By adjusting the mixing amount of PLLA-*b*-PEG block copolymer, micelles with different pH-sensitivity could be obtained. When 10 wt.% PLLA-*b*-PEG was added into PH-*b*-PEG micelles, it did not change significantly in destabilizing pH, and the destabilization of micelles occurred below pH 8.0. However, 25 wt.% addition of PLLA-*b*-PEG significantly improved micellar stability, and destabilizing pH was below pH 7.0. Compared to 25 wt.% addition, the destabilizing pH of mixed micelles with 40 wt.% PLLA-*b*-PEG blend was shifted a bit further downward, which occurred below pH 6.8. It was suggested that the destabilizing pH gradually decreased as the content of PLLA-*b*-PEG block copolymer increased. Additionally, the results for cumulative release of DOX-loaded mixed micelles for 24 h at different pH were presented in Figure 12. At pH 7.0, mixed micelles containing 10 wt.% PLLA-*b*-PEG displayed the highest cumulative release amount (more than 60%). And about 32% of DOX was released from mixed micelles with 25 wt.% or 40 wt.% of PLLA-*b*-PEG. At pH 6.8, three mixed micelles with 10, 25, and 40 wt.% PLLA-*b*-PEG released approximately 75, 70, and 35%, respectively. At pH 5.0, 10 and 25 wt.% PLLA-*b*-PEG mixed micelles both achieved a release of more than 80%, and for 40 wt.% PLLA-b-PEG micelle, the drug release was more than 70%. It is probable that at low pH, the imidazole group is ionized on the polyhistidine chain, accompanied by an increase in the hydrophilicity of the polyhistidine chain. As a result, the hydrophobic environment of mixed micelle was disrupted, and PLLA-*b*-PEG was separated from micelles, leading to micelles disintegration. The added amount of PLLA-*b*-PEG block copolymer had an effect on the pH value of micellar dissociation. These results suggested that appropriate proportion of PLLA-*b*-PEG polymer could improve the discrimination and sensitivity of the mixed micelles to the acidic extracellular pH of the tumoral microenvironment.

In another example, poly(histidine-*co*-phenylalanine) (PHP) was synthesized via the ring-opening polymerization of the benzyl-protected L-histidine NCA and L-phenylalanine NCA (Kim et al., 2008). As the proportion of phenylalanine increased from 10 to 27%, the apparent pK_b_ value of PHP decreased from 6.7 to 4.8. In addition, PHP could be coupled with activated monocarboxylated PEG2000 to form a diblock polymer (PHP-*b*-PEG). Various PHP-*b*-PEG polymers were designed by adjusting the molar percentage of phenylalanine in PHP, including PHP(10)-*b*-PEG, PHP(16)-*b*-PEG, PHP(22)-*b*-PEG, and PHP(27)-*b*-PEG. Above four-block polymers self-assembled to form micelles, which were named as PHSM(10), PHSM(16), PHSM(22), and PHSM(27), respectively. pH-responsiveness of polymeric micelles was evaluated by critical micelle concentration (CMC), particle size, and the light transmittance. In the aspect of CMC, at pH 6.0, CMCs of PHSM(10) and PHSM(16) were above 90 μg ml^−1^. However, when pH was greater than or equal to 6.5, they significantly increased. The elevated CMC of PHSM(10) and PHSM(16) indicated that both micelles were unstable at low pH. CMCs of PHSM(22) and PHSM(27) were lower than 20 μg ml^−1^ at pH 6.0 and 6.5, suggesting that they have good stability. In terms of the change of particle size, PHSM(10) and PHSM(16) showed a great difference. The former decreased rapidly, and the latter exhibited a gradual decrease in the range of pH 6.0 ∼ 7.4. Nevertheless, the particle size of PHSM(22) and PHSM(27) had an insignificant change at pH 5.5 and 6.5. The transmissivity of micellar solution was correlated with change in particle size. The transmittance transition points of PHSM(10), PHSM(16), PHSM(22), and PHSM(27) decreased successively at pH 7.0, 6.5, 5.8, and 5.2, respectively. These observations indicated that the introduction of hydrophobic comonomers could adjust pH-sensitivity of micellar core based on polyhistidine.

**FIGURE 13:**
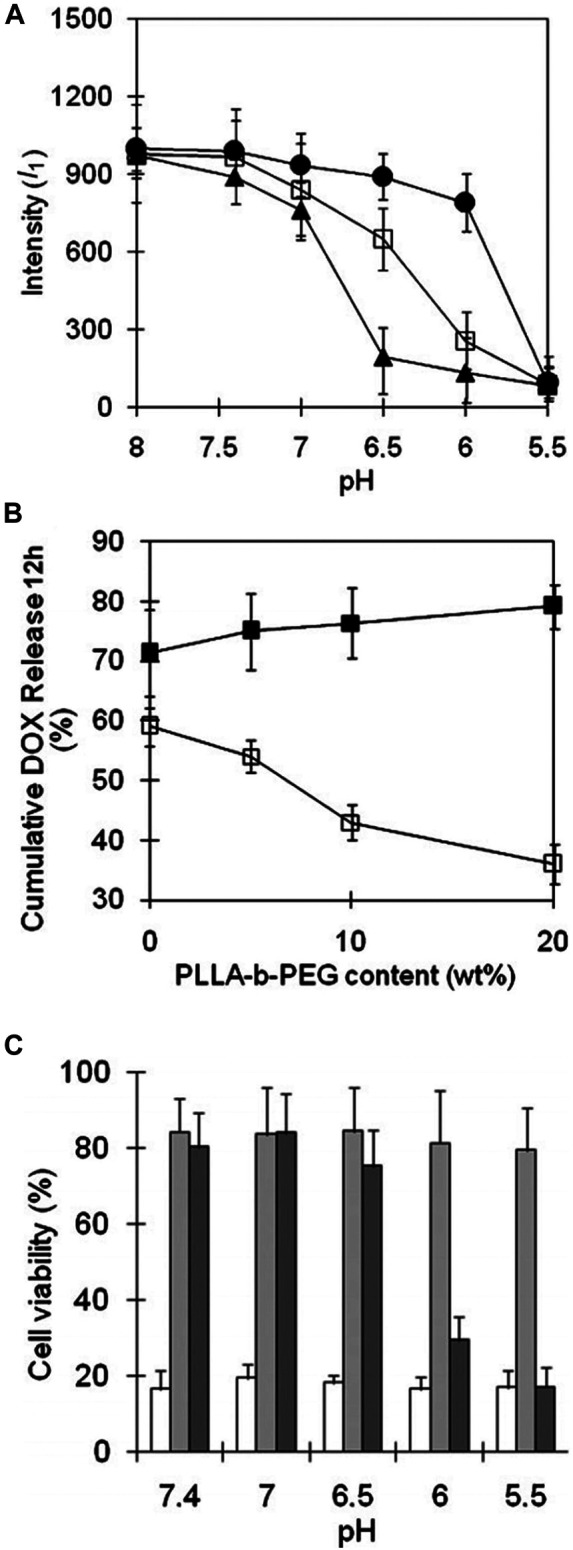
**(A)** The change of pyrene fluorescence intensity (*I*_1_) with pH at a constant micelle concentration (0.05 g L^−1^). m-PHSM constructed with the different amounts of PLLA-b-PEG (5%, ▲; 10%, □; 20%, ●) was monitored in NaOH (or HCl)-Na_2_B_4_O_7_ buffer solution (pH 8.0) with exposure to each pH for 24 h. **(B)** Cumulative amount of DOX for 12 h released from various m-PHSM at pH 6.5 (□) and pH 6.0 (■). **(C)** Cell viabilities determined by MTT assay of ovarian A2780 wild-type carcinoma cells treated with micelles: free DOX (white), DOX/PHIM (gray), and DOX/m-PHSM(20%) (dark gray). DOX dose was equivalent to 1,000 ng ml^−1^ in each formulation (Kim et al., 2008). Abbreviation: m-PHSM, pH-sensitive mixed micelles containing poly(histidine (His)-co-phenylalanine (Phe))-b-poly(ethylene glycol) (PEG) and poly(L-lactic acid) (PLLA)-b-PEG; PHIM, pH-insensitive PLLA-b-PEG micelles.

In the same report, Kim et al. (2008) mixed PHP-*b*-PEG with PLLA(*M*_*n*_ 3,000)-*b*-PEG(*M*_*n*_ 2,000) to design new pH-sensitive micelles with responsiveness to specific pH, which was possible to achieve accurate targeting of tumor organelles. As mentioned above, the destabilizing pH of PHSM(16) was approximately 6.5. When PLLA-*b*-PEG was introduced into PHP(16)-*b*-PEG to form mixed micelles, micellar triggering pH could be modulated by the added ratio of PLLA-*b*-PEG. pH-sensitivity of mixed micelles was determined by the fluorescence intensity of pyrene under different pH conditions, which was shown in Figure 13A. The mixed micelles containing 5, 10, and 20% of PLLA-*b*-PEG were named as m-PHSM (5%), m-PHSM (10%), and m-PHSM (20%), respectively. For m-PHSM (5%) and m-PHSM (10%), the main change in fluorescence intensity occurred at pH > 6.0. The fluorescence intensity of m-PHSM (20%) altered primarily in the range of 5.5 ∼ 6.0, which was closer to that of early endosomes. Obviously, the sensitivity of m-PHSM to low pH increased with increasing proportion of blended PLLA-*b*-PEG. This was also confirmed by the release capacity of DOX-loaded m-PHSM (DOX/m-PHSM) at different pH conditions. The 12-h cumulative DOX release from DOX/m-PHSM at pH 6.0 and 6.5 manifested that release of DOX at pH 6.5 decreased with increasing PLLA-*b*-PEG ratio in micelles and an opposite trend happened at pH 6.0 (Figure 13B). Compared with DOX/m-PHSM (5%) and DOX/m-PHSM (10%), the amount of drug release from DOX/m-PHSM (20%) was the least at pH 6.5, but it was the most at pH 6.0. The rapid release of DOX at pH 6.0 significantly improved the efficacy. In the *in-vitro* cytotoxicity study, DOX/m-PHSM (20%) was incubated with wild-type human ovarian A2780 for 48 h. The cell viability decreased from ∼75% under pH 6.5 to less than 40% under pH 6.0 (Figure 13C). The above results indicated that m-PHSM (20%) was likely to achieve the targeting of early endosome.

## Arginine-Based Micelles

Arginine possessed some special functions, which were used to fabricate micelles, including cell-membrane-penetrating function and arginine-derived nitric oxide.

### Construction of Micelles Utilizing Membrane-Penetrating Function of Arginine

Arginine is a primary component of most cell-penetrating peptides (CPPs), whose guanidine group has a key role in the transmembrane transport of peptides (Schröder et al., 2008; Wender et al., 2008).

**FIGURE 14:**
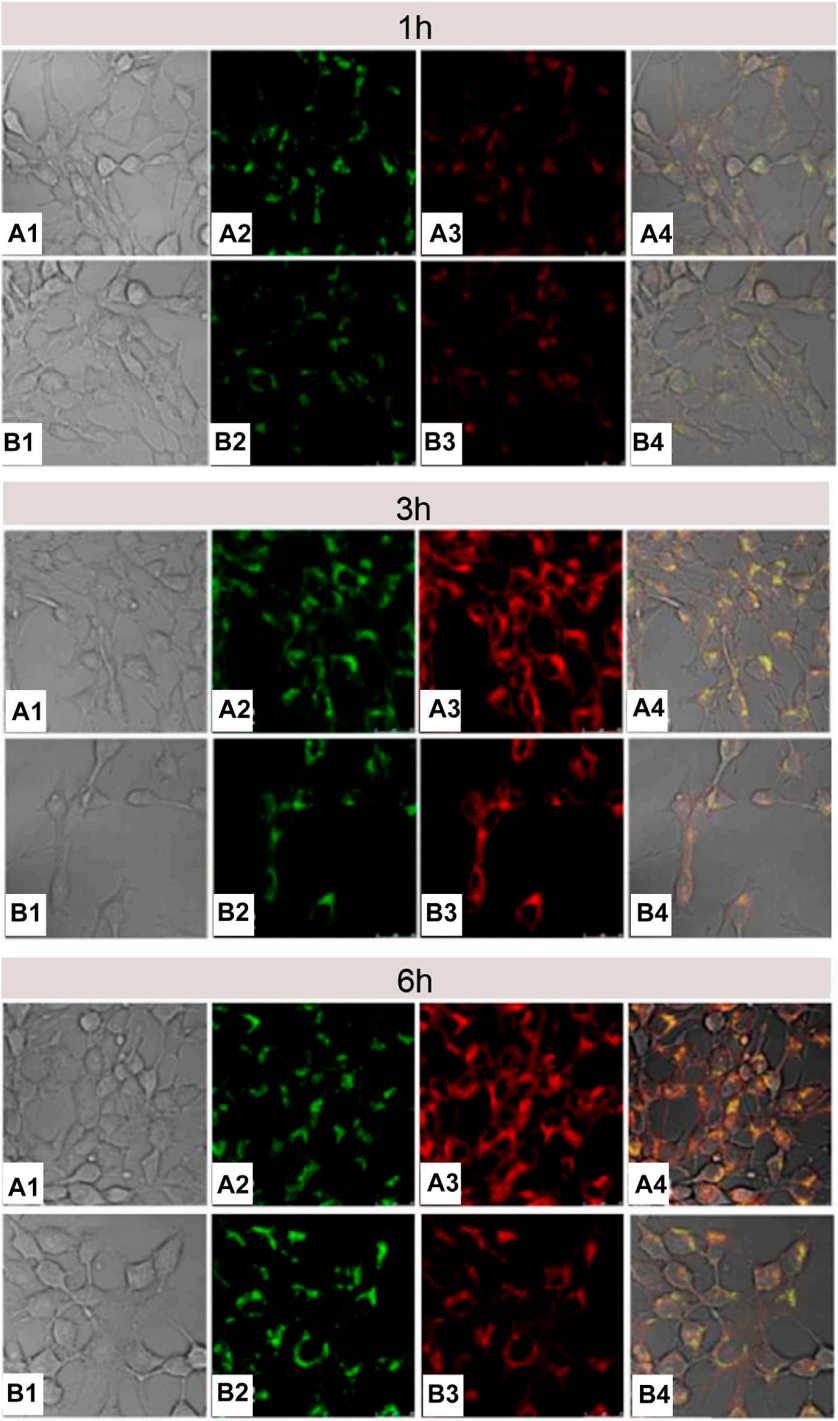
The CLSM images of 4T1 cells treated with DOX/Arg-PEG-PCL-Py **(A)** and DOX/Ac-PEG-PCL-Py micelles **(B)** for 1, 3, and 6 h. The numbers 1, 2, 3, and 4 indicated the bright field, LysoTracker Green fluorescence, DOX fluorescence, and the overlay images, respectively. The concentration of DOX was 10 μg ml^−1^ (Luo et al., 2016). Abbreviation: Arg-PEG-PCL, arginine-modified poly(ethylene glycol)-b-poly(ε-caprolactone); Ac-PEG-PCL, acetic acid-modified PEG-PCL; Py, pyrene.

Cell-membrane-penetrating ability of arginine is related to the quantity of arginine and the density of guanidine groups. Oligoarginine with more than six repeating units of arginine exhibited perfect membrane-penetration effect (Wang P. et al., 2019; Zhai et al., 2019), and vice versa (Mitchell et al., 2000; Wender et al., 2000). In another example, Macewan and Chilkoti (2012) connected five arginines with hydrophobic segment of amphiphilic elastin-like polypeptides (ELP_BC_s) to build Arg_5_-ELP_BC_ with characteristic of temperature sensitivity (Wright and Conticello, 2002; Dreher et al., 2008). Slightly high temperature (e.g. 42°C) could trigger Arg_5_-ELP_BC_ to self-assemble into micelles, thus resulting in the increase of local arginine density. Confocal microscopy displayed that at 37°C, there was no obvious uptake by Hela cells incubated with Arg_5_-ELP_BC_ for 1 h, whereas, at 42°C, the self-assembled Arg_5_-ELP_BC_ micelles showed significantly increased cellular uptake. Moreover, flow cytometry manifested that there was approximately 8-fold difference in cellular uptake for Arg_5_-ELP_BC_ at 37 and 42°C. It could be inferred that it was an opportunity for polymer containing less than six arginines to realize cell-membrane penetration if only the surface of micelles was covered with high density of arginine guanidine groups by some way. Song Luo’s research (Luo et al., 2016) was consistent with above inference. Specifically, single arginine and pyrene (py) were separately attached to the end of PEG and PCL segment in PEG-*b*-PCL to construct Arg-PEG-*b*-PCL-Py copolymer. Next, Arg-PEG-*b*-PCL-Py copolymer self-assembled into micelles, and guanidine groups of arginine were exposed on the surface of them. In comparison with micelles without modifying arginine, arginine-modified micelles had a more efficient endocytosis and more rapid endo-lysosomal escape (Figure 14), indicating that guanidine-functionalized micelles have a stronger capability to pass through the cell membrane. It was important to note that if it was sufficient for local arginine density on micellar surface, even single arginine-decorated micelles could promote tumor cell internalization.

### Construction of Micelles Based on Antitumor Ability of Nitric Oxide Derived From Arginine

As early as the 1980s, the immune function of organisms could be regulated by L-arginine (Hibbs et al., 1987). Later, the inhibitory effect of L-arginine on tumor and its mechanism of action were also gradually revealed. Briefly, L-arginine is the substrate of nitric oxide synthase (NOS) (Iyengar et al., 1987). At the early stage of cancer, M1 macrophages infiltrating into tumor tissues overexpress inducible NO synthase (iNOS), which converted L-arginine to NO. The gaseous and lipophilic NO rapidly permeates tumor tissue from M1 macrophages (Thomas et al., 2008). High concentration of NO is cytotoxic and can cause tumor cell apoptosis or induce tumor cell necrosis (Singh et al., 1996; Ambs et al., 1998; Kudo and Nagasaki, 2015).

**FIGURE 15:**
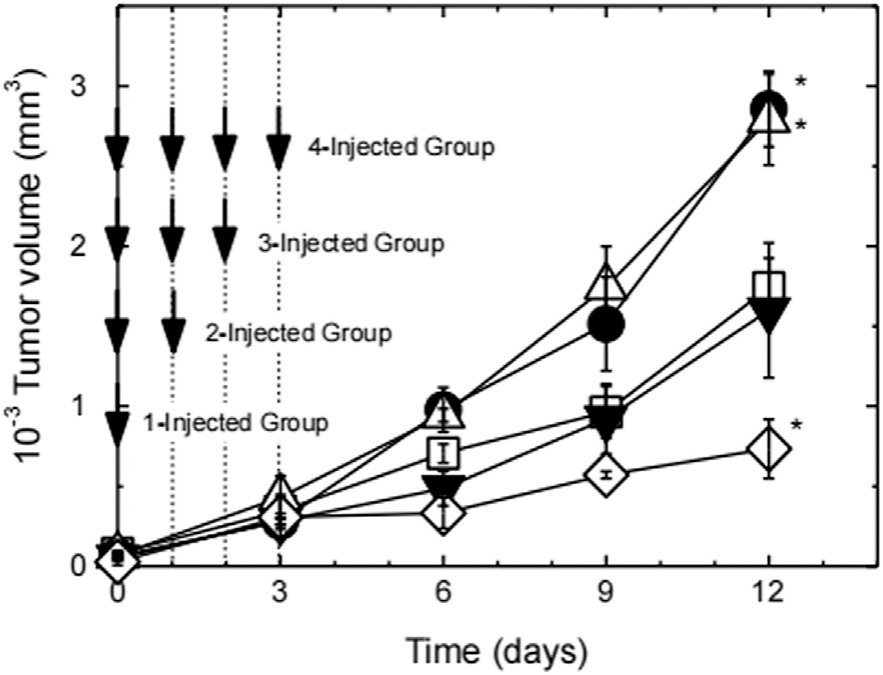
Tumor growth curve of tumor-bearing mice after the first injection of PEG-b-P(L-Arg)/m (Kudo and Nagasaki, 2015). The number of injections was changed from 1 up to 4 times on days 0–3 at a dose of 16 mg/kg on an arginine basis. Injection times are 1 (closed circles), 2 (open triangles), 3 (closed triangles), and 4 (open lozenges). Tumor volume change of PBS-treated mice was also shown as open squares. (*n* = 4, expressed as mean ± S.E., *p < 0.05). Abbreviation: P(L-Arg), poly(L-Arginine); m, micelles.

It has been reported that the increase of L-arginine outside M1 macrophages will upregulate NO (Tsikas et al., 2000). However, systemic administration of free L-arginine is not a good idea, and it is because that there are a lot of problems such as rapid systemic metabolism or excretion, making L-arginine difficult to accumulate at the tumor site (Lind, 2004). But these issues can be solved *via* the “introducing arginine into nano-micelles” strategy. Owing to the enhanced permeability and retention (EPR) effect of tumor, arginine-rich nano-micelles enabled improved arginine accumulation at the tumor site by passive targeting strategy and generated site-specific high concentration of NO to prevent tumor progression. For instance, Kudo and Nagasaki (2015) synthesized cationic poly((ethylene glycol)-*block*-poly (L-arginine) (PEG-*b*-P(L-Arg)) block copolymers with 62 arginine repeating units, which were electrostatically coupled with polyanion chondroitin sulfate (CS) to form a polyionic complex (PIC) micelle with a particle size of about 50 nm and a near-neutral surface charge (average zeta potential: ∼0.09 mV). RAW264.7 macrophages were activated by lipopolysaccharide, which induced the expression of iNOS. The results of *in vivo* distribution of carriers in tumor-bearing mice confirmed that PIC micelles tended to accumulate in the tumor site, which realized the effective delivery of arginine. Subsequently, to investigate *in vivo* antitumor activity of PIC micelles (Figure 15), tumor-bearing mice were divided into four groups, which were named as 1-injected group, 2-injected group, 3-injected group, and 4-injected group, respectively. In 1-injected group, tumor-bearing mice received a 16 mg/kg dose of L-arginine by tail vein injection for one time (on day 0), which existed in the PIC micelles. In 2-injected group, the PIC micelles were administered at 16 mg/kg on L-arginine basis for two times (on days 0 and 1, at a total dose of 32 mg/kg). According to the above-mentioned prescription rules, tumor-bearing mice of 3-injected and 4-injected groups were given a corresponding dose of L-arginine. After 12 d in comparison with the control group, there were significant differences in tumor size for four groups. Tumor volume of 1-injected and 2-injected groups was significantly higher than that of the PBS control group (*p* < 0.05), whereas tumor volume of 3-injected group was close to that of the control group. It was noteworthy that suppression of tumor growth was obviously observed in 4-injected group. These results were also confirmed by Thomas et al. (Hofseth et al., 2003; Thomas et al., 2004). These results showed that the antitumor effect of NO depended on its concentration. Low-dose NO promoted tumor growth, whereas high-dose one inhibited tumor growth. The one possible reason is that low concentration of NO could enhance angiogenesis, thus promoting tumor growth. In contrast, high concentration of NO could lead to tumor cell apoptosis, along with inhibiting tumor growth. Therefore, it is helpful for designing arginine-rich micelles to enhance the therapeutic effect of tumor.

## Conclusion

Material safety is one of the main problems that affect the development of micelles from basic research to clinical application. Raw materials for building micelles were derived from various natural and synthetic materials. Compared to synthetic materials, natural materials have almost no side effect on human body, which become the first choice for micelles. In natural materials, basic amino acids have been paid much attention for non-toxicity and good biocompatibility.

This review shows design ideas for the construction of micelles by using basic amino acids and their derivatives. It can be summarized as follows:

1) Lysine and its derivatives can build the linker, core, and shell of micelles. As linker, lysine, lysine-based dendrimer, and polylysine provide the rich active groups to connect hydrophilic or hydrophobic materials. Additionally, the surplus amino groups of polylysine as linker can load drug by electrostatic interaction or are covalently cross-linked by introducing disulfide bond, thus improving the stability of drug-loaded micelles. In the construction of micellar core, polylysine can be physically combined with negatively charged polymer by electrostatic interaction, or chemically modified in the side chain amino groups to form micellar core. Modification methods for side-chain amino group of polylysine include charge reversal of polylysine by introducing carboxyl groups, cross-linking of polylysine by introducing sulfhydryl groups, and hydrophobic modification of polylysine by introducing hydrophobic molecules. When acting as the shell of micelles, the positive charge of polylysine is shielded by introducing drug, anionic substances, or polymers to temporarily occupy the amino groups, thereby reducing or reversing the charge of drug-loaded micelles, improving their stability in the blood circulation, and enhancing their efficiency of endocytosis by target cells.
2) Both histidine and polyhistidine can act as the core of micelles to load drug and trigger drug release by acidic pH. When introducing hydrophobic material into the micellar core based on polyhistidine, either by mixing hydrophobic polymer blocks or by introducing other hydrophobic components into the polyhistidine blocks, the ability of micelles to recognize small differences in pH is improved thus achieving accurate targeting of tumor organelles. In addition, polyhistidine as an intermediate layer of triblock polymer micelles can control drug release rate by responding to acidic pH. There is a correlation between the chain length of polyhistidine as the middle layer of micelles and pH-dependent drug release rate.
3) The micelles modified by both single arginine and oligoarginine have high membrane-penetrating ability due to covering the outside of micelles with high density of arginine guanidine groups. Moreover, the high concentration of arginine-rich micelles in tumor site can generate high concentration of NO to enhance antitumor effect.

Considerable progress has been made in the construction of drug-loaded micelles based on lysine, histidine, and arginine, and efficient drug delivery at the target site has been achieved. In addition, cytotoxicity and intracellular delivery processes have been widely studied in many researches. Nevertheless, there are still the following problems to be solved: 1) micelles based on basic amino acids are still produced on a laboratory scale, and issues related to low yield and high cost need to be resolved; 2) most of the researches in drug delivery are only at the basic research stage of cell and animal models. Information was limited on biodistribution, metabolism, and degradation mechanisms of amino acid-based micelles. In order to obtain better biomedical applications, the further corresponding studies related to the above unsolved issues are necessary.

## Data Availability

The original contributions presented in the study are included in the article/supplementary material; further inquiries can be directed to the corresponding author.
